# Tris(tetra­butyl­ammonium) tris­(nitrato-κ^2^ *O*,*O*′)tetra­kis­(thio­cyanato-κ*N*)thorium(IV)

**DOI:** 10.1107/S1600536811009792

**Published:** 2011-03-26

**Authors:** M. Janeth Lozano-Rodriguez, Pierre Thuéry, Sébastien Petit, Roy Copping, Jose Mustre de Leon, Christophe Den Auwer

**Affiliations:** a CEA, Nuclear Energy Division, Radio Chemistry and Processes Department, F-30207 Bagnols sur Cèze, France; b Departamento de Fisica Aplicada, Cinvestav-Merida, Merida, Yuc. 97310, Mexico; c CEA, IRAMIS, UMR 3299 CEA/CNRS, SIS2M, LCCEf, Bat. 125, F-91191 Gif-sur-Yvette, France

## Abstract

The title compound, (C_16_H_36_N)_3_[Th(NCS)_4_(NO_3_)_3_], was obtained from the reaction of Th(NO_3_)_4_·5H_2_O with (Bu_4_N)(NCS). The Th^IV^ atom is in a ten-coordinate environment of irregular geometry, being bound to the N atoms of the four thio­cyanate ions and to three bidentate nitrate ions. The average Th—N and Th—O bond lengths are 2.481 (10) and 2.57 (3) Å, respectively.

## Related literature

For the structures of the parent lanthanide complexes, see: Mullica *et al.* (1997 ▶, 1998 ▶); Farmer *et al.* (2000 ▶). For the structures of related actinide thio­cyanate complexes, see: Countryman & McDonald (1971 ▶); Al-Kazzaz *et al.* (1972 ▶); Charpin *et al.* (1983 ▶); Budantseva *et al.* (2003 ▶). For a description of the Cambridge Structural Database, see: Allen (2002 ▶).
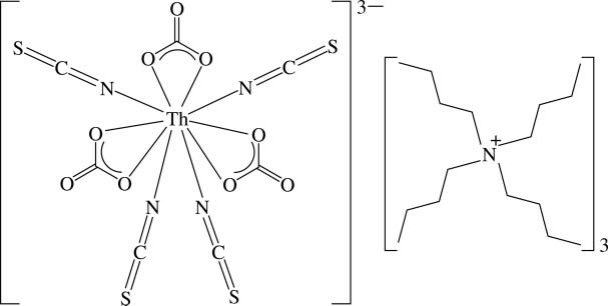


## Experimental

### 

#### Crystal data

(C_16_H_36_N)_3_[Th(NCS)_4_(NO_3_)_3_]
*M* *_r_* = 1377.76
Monoclinic, 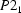

*a* = 12.1057 (7) Å
*b* = 17.5943 (8) Å
*c* = 16.7084 (8) Å
β = 95.946 (3)°
*V* = 3539.6 (3) Å^3^
*Z* = 2
Mo *K*α radiation
μ = 2.28 mm^−1^
*T* = 150 K
0.12 × 0.12 × 0.07 mm

#### Data collection

Nonius KappaCCD area-detector diffractometer
Absorption correction: multi-scan (*SCALEPACK*; Otwinowski & Minor, 1997 ▶) *T*_min_ = 0.703, *T*_max_ = 0.853
170199 measured reflections
13392 independent reflections
11074 reflections with *I* > 2σ(*I*)
*R*_int_ = 0.046

#### Refinement

*R*[*F* ^2^ > 2σ(*F* ^2^)] = 0.037
*wR*(*F* ^2^) = 0.089
*S* = 1.04
13392 reflections
698 parameters
6 restraints
H-atom parameters constrained
Δρ_max_ = 0.98 e Å^−3^
Δρ_min_ = −0.76 e Å^−3^
Absolute structure: Flack (1983 ▶), 6435 Friedel pairs
Flack parameter: −0.008 (5)

### 

Data collection: *COLLECT* (Nonius, 1998 ▶); cell refinement: *HKL-2000* (Otwinowski & Minor, 1997 ▶); data reduction: *HKL-2000*; program(s) used to solve structure: *SHELXS97* (Sheldrick, 2008 ▶); program(s) used to refine structure: *SHELXL97* (Sheldrick, 2008 ▶); molecular graphics: *SHELXTL* (Sheldrick, 2008 ▶); software used to prepare material for publication: *SHELXTL*.

## Supplementary Material

Crystal structure: contains datablocks I, global. DOI: 10.1107/S1600536811009792/tk2728sup1.cif

Structure factors: contains datablocks I. DOI: 10.1107/S1600536811009792/tk2728Isup2.hkl

Additional supplementary materials:  crystallographic information; 3D view; checkCIF report

## Figures and Tables

**Table:** 

Th—N1	2.480 (5)
Th—N2	2.465 (7)
Th—N3	2.488 (5)
Th—N4	2.490 (7)
Th—O1	2.618 (5)
Th—O2	2.563 (4)
Th—O4	2.557 (4)
Th—O5	2.570 (4)
Th—O7	2.615 (6)
Th—O8	2.524 (5)

**Table:** 

O1—Th—O2	49.51 (14)
O4—Th—O5	49.30 (13)
O7—Th—O8	49.44 (15)

**Fig. 1.**
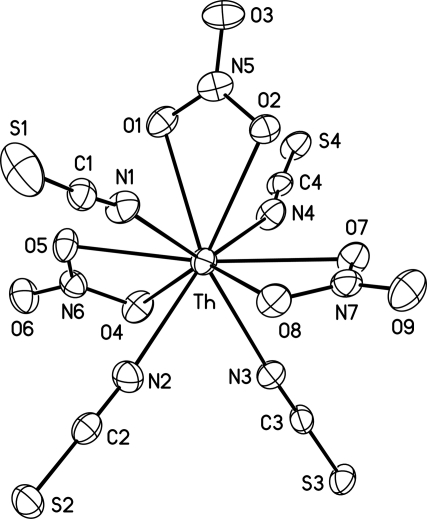
A view of the anion in compound (I) with the atom-numbering scheme. Displacement ellipsoids are drawn at the 20% probability level. H atoms have been omitted.

## supplementary crystallographic information

### Comment

The structures of the parent lanthanide complexes [NBu_4_]_3_[Ce^III^(NCS)_2_(NO_3_)_4_] (Mullica *et al.*, 1998) and [NBu_4_]_3_[Nd^III^(NCS)_2_(NO_3_)_4_] (Farmer *et al.*, 2000) have been determined. In these complexes, the lanthanide cation is ten-coordinated, with two N atoms from the thiocyanate groups and eight O atoms from the bidentate nitrate groups being coordinated. In the case of [NBu_4_]_3_[Yb^III^(NCS)_4_(NO_3_)_2_], the metal is eight-coordinated in agreement with the steric constraints due to lanthanidic contraction. Finally, [N(nC_4_H_9_)_4_]_3_[Lu^III^(NCS)_6_] exhibits an unusual octahedral coordination with 6 thiocyanates around the Lu cation (Mullica *et al.*, 1997). In the mid-1960's, interest in the crystallochemistry of actinide thiocyanate complexes arose. At the beginning of the 1970's, the crystal structure of tetraethyl-ammonium-octathiocyanato-N-uranate(IV) [N(C_2_H_5_)_4_]_4_[U^IV^(NCS)_8_] was determined, and it exhibits eight thiocyanate ligands that are coordinated *via* the nitrogen atom and arranged in the vertices of a cube (Countryman & McDonald, 1971). The corresponding complexes of Th, Pa, Np and Pu were reported to be isostructural but no single-crystal data were determined (Al-Kazzaz *et al.*, 1972). In 1983, the [N(C_2_H_5_)_4_]_4_[Th^IV^(NCS)_8_] complex was reported to display the same cubic coordination than the uranate complex (Charpin *et al.*,  1983). Finally, in 1983, the neptunium adduct [N(CH_3_)_4_]_4_[Np^IV^(NCS)_8_] was reported to be similar to the previously described [N(C_2_H_5_)_4_]_4_[U(NCS)_8_] complex, although the coordination polyhedra are significantly different: in the uranium compound the 8 thiocyanate ligands form a distorted cube (U–N = 2.38 Å) whereas in the neptunium compound they form a distorted tetragonal antiprism (Np–N = 2.39–2.42 Å) (Budantseva *et al.*, 2003).

The family of actinide thiocyanate derivatives is of potential interest for the comprehensive coordination chemistry of f-block elements with applications in selective reactivity. In comparison with parent lanthanide elements, actinide thiocyanate derivatives have been poorly studied in the past. In the title complex [NBu_4_]_3_[Th(NCS)_4_(NO_3_)_3_], (I), the metal cation is 10-coordinated with three bidentate nitrate ions and four N-bonded thiocyanates. The coordination polyhedron is quite irregular, but, if the three nitrate ions are considered as single donors, it may be noted that the four N atoms of the thiocyanate ions and the atom N5 of the nitrate ion bound through O1 and O2 define a mean plane with an r.m.s. deviation of 0.318 Å, which contains the Th atom, displaced by 0.003 (3) Å from it. The other two nitrate ions are on either side of the plane, with a N6···Th···N7 angle of 165.34 (16) °. The average Th–N and Th–O bond lengths of 2.481 (10) and 2.57 (3) Å, respectively, are in agreement with the average values from analogous structures in the Cambridge Structural Database (Version 5.32; Allen, 2002), i.e. 2.48 (3) and 2.58 (5) Å, for 5 and 27 hits, respectively.

### Experimental

Caution! With thorium being a radioactive and chemically toxic element, thorium-containing samples must be handled with suitable care and protection. All starting materials used in these synthetic reactions are available commercially and were used as obtained from the supplier. Th(NO_3_)_4_.5H_2_O (0.474 mmol, 0.27 g) was dissolved in hot ethanol (25 ml). After complete dissolution, tetrabutylammonium thiocyanate, C_17_H_36_N_2_S, (5 mmol, 1.5 g) was added directly to the solution in a ratio Th(IV):SCN^-^ 1:10. The colourless solution was refluxed with stirring for 8 h. The resulting solution was left undisturbed at room temperature and colourless crystals were obtained within three days. The crystals were isolated and washed with cold ethanol. IR results (cm^-1^): ν(CH) 2962.16, 2874.55; ν(CN from SCN) 2040.99; ν(NO) 1278.73, 883.20; ν(CS) 744.29.

### Refinement

Restraints were applied for five bond lengths in the tetrabutylammonium cations. The H atoms were introduced at calculated positions as riding atoms, with C–H bond lengths of 0.97 Å (CH_2_) or 0.96 Å (CH_3_), and with *U*_iso_(H) values of 1.2 (CH_2_) or 1.5 (CH_3_) times *U*_eq_(C).

### Crystal data

**Table:**

(C_16_H_36_N)_3_[Th(NCS)_4_(NO_3_)_3_]	*F*(000) = 1432
*M**_r_* = 1377.76	*D*_x_ = 1.293 Mg m^−^^3^
Monoclinic, *P*2_1_	Mo *K*α radiation, λ = 0.71073 Å
Hall symbol: P 2yb	Cell parameters from 170199 reflections
*a* = 12.1057 (7) Å	θ = 3.2–25.7°
*b* = 17.5943 (8) Å	µ = 2.28 mm^−^^1^
*c* = 16.7084 (8) Å	*T* = 150 K
β = 95.946 (3)°	Irregular, colourless
*V* = 3539.6 (3) Å^3^	0.12 × 0.12 × 0.07 mm
*Z* = 2	

### Data collection

**Table:**

Nonius KappaCCD area-detector diffractometer	13392 independent reflections
Radiation source: fine-focus sealed tube	11074 reflections with *I* > 2σ(*I*)
graphite	*R*_int_ = 0.046
two φ and eleven ω scans with 2° steps (649 frames)	θ_max_ = 25.7°, θ_min_ = 4.1°
Absorption correction: multi-scan (*SCALEPACK*; Otwinowski & Minor, 1997)	*h* = −14→14
*T*_min_ = 0.703, *T*_max_ = 0.853	*k* = −21→21
170199 measured reflections	*l* = −20→20

### Refinement

**Table:**

Refinement on *F*^2^	Secondary atom site location: difference Fourier map
Least-squares matrix: full	Hydrogen site location: inferred from neighbouring sites
*R*[*F*^2^ > 2σ(*F*^2^)] = 0.037	H-atom parameters constrained
*wR*(*F*^2^) = 0.089	*w* = 1/[σ^2^(*F*_o_^2^) + (0.0348*P*)^2^ + 4.0973*P*] where *P* = (*F*_o_^2^ + 2*F*_c_^2^)/3
*S* = 1.04	(Δ/σ)_max_ = 0.002
13392 reflections	Δρ_max_ = 0.98 e Å^−^^3^
698 parameters	Δρ_min_ = −0.76 e Å^−^^3^
6 restraints	Absolute structure: Flack (1983), 6435 Friedel pairs
Primary atom site location: heavy-atom method	Flack parameter: −0.008 (5)

### Special details

**Table:**

Experimental. crystal-to-detector distance 30 mm
Geometry. All e.s.d.'s are estimated using the full covariance matrix. The cell e.s.d.'s are taken into account individually in the estimation of e.s.d.'s in distances, angles and torsion angles; correlations between e.s.d.'s in cell parameters are only used when they are defined by crystal symmetry.
Refinement. Refinement of *F*^2^ against ALL reflections. The weighted *R*-factor *wR* and goodness of fit *S* are based on *F*^2^, conventional *R*-factors *R* are based on *F*, with *F* set to zero for negative *F*^2^. The threshold expression of *F*^2^ > σ(*F*^2^) is used only for calculating *R*-factors(gt) *etc*. and is not relevant to the choice of reflections for refinement. *R*-factors based on *F*^2^ are statistically about twice as large as those based on *F*, and *R*- factors based on ALL data will be even larger.

### Fractional atomic coordinates and isotropic or equivalent isotropic displacement parameters (Å^2^)

**Table:**

	*x*	*y*	*z*	*U*_iso_*/*U*_eq_	
Th	0.494552 (16)	0.250260 (18)	0.231036 (12)	0.05183 (7)	
S1	0.8148 (2)	0.09946 (16)	0.3932 (2)	0.1219 (11)	
S2	0.4103 (2)	−0.03910 (11)	0.26535 (13)	0.0803 (6)	
S3	0.17368 (14)	0.22589 (11)	−0.00503 (10)	0.0718 (6)	
S4	0.34264 (19)	0.52512 (11)	0.21889 (12)	0.0778 (6)	
O1	0.5736 (4)	0.3482 (3)	0.3402 (3)	0.0666 (12)	
O2	0.6625 (4)	0.3393 (2)	0.2343 (3)	0.0597 (11)	
O3	0.7191 (5)	0.4203 (3)	0.3291 (4)	0.0858 (16)	
O4	0.3034 (3)	0.2585 (5)	0.2829 (3)	0.0724 (14)	
O5	0.4354 (3)	0.2291 (3)	0.3727 (2)	0.0661 (16)	
O6	0.2663 (4)	0.2292 (3)	0.4035 (3)	0.0840 (19)	
O7	0.5662 (5)	0.2889 (3)	0.0944 (3)	0.0688 (15)	
O8	0.6172 (4)	0.1797 (3)	0.1428 (3)	0.0688 (13)	
O9	0.6818 (5)	0.2177 (3)	0.0337 (3)	0.0942 (18)	
N1	0.6508 (5)	0.1905 (4)	0.3162 (4)	0.0662 (15)	
N2	0.4402 (6)	0.1159 (4)	0.2416 (4)	0.0707 (18)	
N3	0.3500 (5)	0.2278 (3)	0.1164 (3)	0.0693 (18)	
N4	0.4190 (6)	0.3796 (4)	0.1990 (4)	0.0686 (17)	
N5	0.6536 (5)	0.3704 (4)	0.3031 (4)	0.0665 (15)	
N6	0.3322 (4)	0.2384 (4)	0.3553 (3)	0.0603 (16)	
N7	0.6217 (5)	0.2291 (3)	0.0884 (4)	0.0616 (17)	
N8	0.6507 (5)	0.9468 (3)	0.0700 (3)	0.0554 (16)	
N9	0.0172 (5)	0.8172 (4)	0.7822 (4)	0.0602 (17)	
N10	0.4640 (5)	0.0695 (3)	0.5465 (3)	0.0586 (13)	
C1	0.7183 (6)	0.1502 (4)	0.3480 (4)	0.0594 (16)	
C2	0.4261 (6)	0.0508 (5)	0.2514 (4)	0.0595 (17)	
C3	0.2766 (5)	0.2270 (3)	0.0657 (4)	0.0605 (19)	
C4	0.3867 (6)	0.4403 (4)	0.2094 (4)	0.0560 (16)	
C5	0.6163 (6)	0.8641 (3)	0.0603 (4)	0.0587 (16)	
H5A	0.6823	0.8328	0.0699	0.070*	
H5B	0.5858	0.8560	0.0049	0.070*	
C6	0.5313 (7)	0.8369 (4)	0.1155 (4)	0.072 (2)	
H6A	0.5530	0.8543	0.1700	0.086*	
H6B	0.4592	0.8585	0.0978	0.086*	
C7	0.5235 (6)	0.7516 (7)	0.1145 (4)	0.0810 (19)	
H7A	0.5952	0.7300	0.1337	0.097*	
H7B	0.5040	0.7342	0.0598	0.097*	
C8	0.4353 (9)	0.7240 (5)	0.1683 (7)	0.117 (4)	
H8A	0.4553	0.7404	0.2227	0.176*	
H8B	0.4314	0.6695	0.1668	0.176*	
H8C	0.3642	0.7448	0.1490	0.176*	
C9	0.5505 (7)	0.9995 (4)	0.0598 (5)	0.067 (2)	
H9A	0.5037	0.9888	0.1022	0.081*	
H9B	0.5763	1.0515	0.0666	0.081*	
C10	0.4810 (7)	0.9931 (5)	−0.0202 (5)	0.068 (2)	
H10A	0.4555	0.9411	−0.0281	0.082*	
H10B	0.5261	1.0056	−0.0630	0.082*	
C11	0.3824 (8)	1.0457 (6)	−0.0240 (8)	0.106 (3)	
H11A	0.4086	1.0974	−0.0151	0.127*	
H11B	0.3377	1.0328	0.0190	0.127*	
C12	0.3102 (11)	1.0419 (8)	−0.1038 (9)	0.142 (6)	
H12A	0.3443	1.0706	−0.1435	0.213*	
H12B	0.2383	1.0628	−0.0976	0.213*	
H12C	0.3023	0.9899	−0.1209	0.213*	
C13	0.7278 (6)	0.9613 (4)	0.0074 (4)	0.0640 (18)	
H13A	0.6902	0.9470	−0.0445	0.077*	
H13B	0.7915	0.9280	0.0180	0.077*	
C14	0.7700 (6)	1.0420 (4)	0.0011 (4)	0.0676 (18)	
H14A	0.7918	1.0619	0.0545	0.081*	
H14B	0.7112	1.0739	−0.0243	0.081*	
C15	0.8697 (6)	1.0440 (5)	−0.0483 (4)	0.074 (2)	
H15A	0.8522	1.0141	−0.0968	0.089*	
H15B	0.9326	1.0203	−0.0174	0.089*	
C16	0.9033 (8)	1.1249 (6)	−0.0726 (7)	0.087 (3)	
H16A	0.8446	1.1467	−0.1083	0.130*	
H16B	0.9698	1.1222	−0.0992	0.130*	
H16C	0.9165	1.1559	−0.0253	0.130*	
C17	0.7096 (8)	0.9606 (5)	0.1533 (4)	0.080 (3)	
H17A	0.6587	0.9484	0.1926	0.096*	
H17B	0.7266	1.0143	0.1586	0.096*	
C18	0.8160 (8)	0.9161 (6)	0.1741 (5)	0.097 (3)	
H18A	0.8015	0.8626	0.1635	0.116*	
H18B	0.8709	0.9327	0.1395	0.116*	
C19	0.8629 (11)	0.9258 (8)	0.2612 (6)	0.174 (7)	
H19A	0.8747	0.9794	0.2725	0.209*	
H19B	0.8094	0.9071	0.2959	0.209*	
C20	0.9715 (11)	0.8837 (10)	0.2800 (9)	0.224 (10)	
H20A	0.9666	0.8348	0.2542	0.336*	
H20B	0.9861	0.8770	0.3371	0.336*	
H20C	1.0306	0.9124	0.2606	0.336*	
C21	−0.0421 (5)	0.7440 (7)	0.8028 (4)	0.0671 (17)	
H21A	−0.0789	0.7526	0.8508	0.081*	
H21B	−0.0989	0.7324	0.7592	0.081*	
C22	0.0341 (9)	0.6752 (5)	0.8168 (6)	0.079 (3)	
H22A	0.0672	0.6640	0.7677	0.095*	
H22B	0.0936	0.6873	0.8582	0.095*	
C23	−0.0271 (8)	0.6052 (6)	0.8421 (6)	0.104 (3)	
H23A	0.0254	0.5638	0.8522	0.125*	
H23B	−0.0586	0.6159	0.8919	0.125*	
C24	−0.1173 (9)	0.5810 (8)	0.7801 (8)	0.140 (5)	
H24A	−0.1811	0.6129	0.7832	0.210*	
H24B	−0.1368	0.5291	0.7894	0.210*	
H24C	−0.0922	0.5855	0.7276	0.210*	
C25	0.0753 (6)	0.8069 (5)	0.7064 (4)	0.0689 (19)	
H25A	0.1053	0.8556	0.6920	0.083*	
H25B	0.1373	0.7724	0.7183	0.083*	
C26	0.0016 (5)	0.7765 (4)	0.6342 (4)	0.068 (2)	
H26A	−0.0690	0.8029	0.6290	0.082*	
H26B	−0.0124	0.7227	0.6415	0.082*	
C27	0.0615 (7)	0.7887 (5)	0.5573 (4)	0.083 (2)	
H27A	0.0699	0.8428	0.5482	0.099*	
H27B	0.1350	0.7663	0.5651	0.099*	
C28	−0.0035 (6)	0.7532 (11)	0.4846 (4)	0.105 (2)	
H28A	−0.0001	0.6988	0.4892	0.158*	
H28B	0.0279	0.7688	0.4368	0.158*	
H28C	−0.0795	0.7694	0.4817	0.158*	
C29	−0.0710 (7)	0.8776 (5)	0.7731 (5)	0.082 (2)	
H29A	−0.1022	0.8829	0.8239	0.098*	
H29B	−0.1301	0.8606	0.7335	0.098*	
C30	−0.0323 (10)	0.9547 (6)	0.7481 (6)	0.108 (3)	
H30A	0.0474	0.9529	0.7460	0.129*	
H30B	−0.0471	0.9914	0.7889	0.129*	
C31	−0.0882 (11)	0.9828 (8)	0.6652 (9)	0.156 (5)	
H31A	−0.0672	0.9493	0.6231	0.187*	
H31B	−0.1682	0.9804	0.6650	0.187*	
C32	−0.0552 (13)	1.0620 (8)	0.6475 (10)	0.176 (7)	
H32A	−0.0864	1.0963	0.6837	0.265*	
H32B	−0.0823	1.0746	0.5931	0.265*	
H32C	0.0243	1.0661	0.6542	0.265*	
C33	0.1076 (6)	0.8374 (5)	0.8498 (5)	0.074 (2)	
H33A	0.1625	0.7971	0.8540	0.089*	
H33B	0.1445	0.8834	0.8347	0.089*	
C34	0.0682 (6)	0.8496 (4)	0.9324 (4)	0.0705 (19)	
H34A	0.0108	0.8883	0.9286	0.085*	
H34B	0.0361	0.8028	0.9500	0.085*	
C35	0.1609 (8)	0.8737 (7)	0.9933 (5)	0.105 (3)	
H35A	0.2208	0.8372	0.9925	0.126*	
H35B	0.1887	0.9223	0.9766	0.126*	
C36	0.1336 (9)	0.8812 (8)	1.0780 (6)	0.100 (4)	
H36A	0.0795	0.9207	1.0810	0.150*	
H36B	0.1997	0.8938	1.1122	0.150*	
H36C	0.1042	0.8340	1.0952	0.150*	
C37	0.5315 (7)	−0.0021 (4)	0.5596 (5)	0.0698 (19)	
H37A	0.5858	0.0052	0.6059	0.084*	
H37B	0.4825	−0.0430	0.5725	0.084*	
C38	0.5913 (9)	−0.0263 (5)	0.4895 (6)	0.089 (3)	
H38A	0.6478	0.0107	0.4798	0.107*	
H38B	0.5395	−0.0303	0.4413	0.107*	
C39	0.6447 (8)	−0.1034 (5)	0.5101 (6)	0.091 (3)	
H39A	0.6977	−0.0983	0.5574	0.109*	
H39B	0.5878	−0.1391	0.5226	0.109*	
C40	0.7034 (12)	−0.1344 (7)	0.4410 (8)	0.140 (4)	
H40A	0.6515	−0.1383	0.3937	0.211*	
H40B	0.7331	−0.1838	0.4550	0.211*	
H40C	0.7627	−0.1008	0.4307	0.211*	
C41	0.3767 (7)	0.0594 (6)	0.4756 (5)	0.072 (2)	
H41A	0.3289	0.1038	0.4722	0.087*	
H41B	0.4138	0.0578	0.4268	0.087*	
C42	0.3047 (8)	−0.0106 (7)	0.4780 (5)	0.113 (4)	
H42A	0.3475	−0.0548	0.4651	0.136*	
H42B	0.2845	−0.0172	0.5322	0.136*	
C43	0.2008 (8)	−0.0071 (7)	0.4208 (6)	0.125 (4)	
H43A	0.2180	0.0142	0.3700	0.150*	
H43B	0.1724	−0.0581	0.4107	0.150*	
C44	0.1158 (10)	0.0395 (9)	0.4535 (8)	0.173 (6)	
H44A	0.1071	0.0234	0.5074	0.260*	
H44B	0.0465	0.0338	0.4205	0.260*	
H44C	0.1381	0.0919	0.4540	0.260*	
C45	0.4096 (6)	0.0820 (4)	0.6231 (4)	0.0636 (18)	
H45A	0.3616	0.0390	0.6303	0.076*	
H45B	0.4674	0.0820	0.6679	0.076*	
C46	0.3425 (7)	0.1532 (6)	0.6281 (5)	0.084 (2)	
H46A	0.2874	0.1570	0.5818	0.101*	
H46B	0.3904	0.1974	0.6290	0.101*	
C47	0.2859 (8)	0.1495 (5)	0.7048 (5)	0.099 (3)	
H47A	0.2404	0.1041	0.7029	0.118*	
H47B	0.3427	0.1441	0.7498	0.118*	
C48	0.2151 (11)	0.2156 (8)	0.7206 (8)	0.184 (8)	
H48A	0.2569	0.2616	0.7172	0.276*	
H48B	0.1913	0.2111	0.7734	0.276*	
H48C	0.1514	0.2167	0.6813	0.276*	
C49	0.5345 (6)	0.1377 (4)	0.5282 (4)	0.0653 (18)	
H49A	0.4862	0.1813	0.5173	0.078*	
H49B	0.5697	0.1271	0.4798	0.078*	
C50	0.6245 (7)	0.1583 (4)	0.5958 (5)	0.075 (2)	
H50A	0.5925	0.1649	0.6462	0.090*	
H50B	0.6805	0.1188	0.6026	0.090*	
C51	0.6745 (10)	0.2320 (9)	0.5699 (6)	0.148 (6)	
H51A	0.7169	0.2224	0.5248	0.177*	
H51B	0.6154	0.2673	0.5523	0.177*	
C52	0.7486 (11)	0.2670 (8)	0.6372 (9)	0.215 (9)	
H52A	0.7070	0.2759	0.6822	0.323*	
H52B	0.7771	0.3144	0.6196	0.323*	
H52C	0.8092	0.2332	0.6530	0.323*	

### Atomic displacement parameters (Å^2^)

**Table:**

	*U*^11^	*U*^22^	*U*^33^	*U*^12^	*U*^13^	*U*^23^
Th	0.05059 (10)	0.05223 (10)	0.05088 (10)	0.00249 (19)	−0.00323 (7)	0.00516 (19)
S1	0.0680 (14)	0.115 (2)	0.180 (3)	0.0203 (13)	0.0041 (16)	0.076 (2)
S2	0.1165 (18)	0.0563 (11)	0.0730 (13)	−0.0104 (11)	0.0329 (12)	−0.0032 (9)
S3	0.0569 (9)	0.1043 (18)	0.0523 (9)	−0.0091 (9)	−0.0034 (7)	0.0080 (8)
S4	0.1015 (15)	0.0605 (11)	0.0671 (12)	0.0195 (10)	−0.0124 (11)	−0.0097 (9)
O1	0.059 (3)	0.085 (3)	0.056 (3)	0.013 (3)	0.004 (2)	−0.014 (2)
O2	0.059 (3)	0.061 (3)	0.060 (3)	0.003 (2)	0.007 (2)	−0.008 (2)
O3	0.079 (4)	0.064 (3)	0.111 (4)	0.002 (3)	−0.003 (3)	−0.031 (3)
O4	0.056 (2)	0.088 (4)	0.070 (3)	0.023 (3)	−0.0072 (19)	0.000 (4)
O5	0.050 (2)	0.095 (5)	0.053 (2)	0.007 (2)	0.0033 (18)	0.019 (2)
O6	0.067 (3)	0.102 (6)	0.087 (3)	−0.001 (3)	0.026 (3)	0.017 (3)
O7	0.090 (4)	0.054 (3)	0.062 (3)	0.020 (3)	0.004 (3)	−0.005 (2)
O8	0.081 (3)	0.053 (3)	0.070 (3)	0.015 (2)	0.000 (3)	−0.003 (2)
O9	0.099 (4)	0.115 (5)	0.071 (3)	0.017 (3)	0.021 (3)	−0.024 (3)
N1	0.056 (3)	0.068 (4)	0.070 (4)	0.014 (3)	−0.013 (3)	0.003 (3)
N2	0.073 (4)	0.061 (4)	0.075 (4)	−0.001 (3)	−0.004 (4)	0.012 (4)
N3	0.068 (3)	0.079 (5)	0.058 (3)	−0.010 (3)	−0.009 (3)	0.006 (3)
N4	0.080 (4)	0.062 (4)	0.060 (4)	0.022 (3)	−0.006 (3)	0.001 (3)
N5	0.059 (4)	0.061 (4)	0.079 (4)	0.014 (3)	0.003 (3)	−0.017 (3)
N6	0.056 (3)	0.054 (5)	0.071 (3)	0.002 (3)	0.005 (3)	0.008 (3)
N7	0.076 (4)	0.047 (4)	0.059 (3)	−0.004 (3)	−0.003 (3)	−0.005 (3)
N8	0.070 (4)	0.055 (3)	0.042 (3)	−0.007 (3)	0.009 (3)	−0.015 (3)
N9	0.050 (3)	0.076 (4)	0.054 (4)	0.000 (3)	0.005 (3)	0.000 (3)
N10	0.066 (3)	0.064 (3)	0.045 (3)	−0.005 (3)	0.004 (3)	0.009 (3)
C1	0.048 (4)	0.074 (5)	0.057 (4)	−0.004 (3)	0.007 (3)	0.008 (3)
C2	0.056 (4)	0.075 (5)	0.049 (4)	0.008 (4)	0.010 (3)	−0.008 (4)
C3	0.055 (3)	0.072 (5)	0.055 (4)	−0.010 (3)	0.006 (3)	0.016 (3)
C4	0.052 (4)	0.063 (4)	0.051 (4)	0.000 (3)	−0.007 (3)	−0.001 (3)
C5	0.076 (5)	0.045 (3)	0.055 (4)	−0.001 (3)	0.009 (3)	−0.009 (3)
C6	0.100 (6)	0.049 (4)	0.067 (5)	−0.013 (4)	0.019 (4)	−0.004 (3)
C7	0.114 (5)	0.062 (4)	0.064 (4)	−0.022 (7)	−0.007 (3)	0.015 (7)
C8	0.135 (8)	0.091 (7)	0.126 (8)	−0.032 (6)	0.015 (7)	0.020 (5)
C9	0.087 (6)	0.050 (4)	0.068 (5)	−0.008 (4)	0.026 (4)	−0.013 (4)
C10	0.079 (7)	0.051 (4)	0.075 (7)	−0.004 (4)	0.008 (5)	0.000 (5)
C11	0.086 (7)	0.065 (5)	0.166 (11)	0.017 (5)	0.008 (7)	0.010 (7)
C12	0.111 (9)	0.128 (11)	0.179 (15)	0.031 (8)	−0.022 (10)	0.058 (11)
C13	0.079 (5)	0.073 (4)	0.041 (3)	−0.005 (4)	0.011 (3)	−0.010 (3)
C14	0.079 (5)	0.076 (5)	0.049 (4)	−0.012 (4)	0.012 (3)	−0.005 (3)
C15	0.062 (4)	0.107 (6)	0.052 (4)	−0.002 (4)	0.007 (3)	−0.003 (4)
C16	0.071 (6)	0.104 (7)	0.085 (6)	−0.024 (5)	0.010 (5)	−0.008 (5)
C17	0.117 (7)	0.086 (6)	0.041 (4)	−0.024 (5)	0.019 (4)	−0.011 (4)
C18	0.120 (8)	0.108 (7)	0.056 (5)	−0.029 (6)	−0.015 (5)	0.011 (4)
C19	0.212 (15)	0.229 (15)	0.069 (7)	−0.105 (13)	−0.041 (8)	0.016 (8)
C20	0.173 (13)	0.31 (2)	0.162 (13)	−0.091 (14)	−0.120 (11)	0.133 (14)
C21	0.055 (3)	0.088 (5)	0.061 (3)	0.002 (6)	0.014 (3)	−0.002 (6)
C22	0.103 (7)	0.068 (5)	0.067 (6)	−0.003 (5)	0.011 (6)	−0.010 (5)
C23	0.119 (8)	0.100 (7)	0.093 (7)	0.027 (6)	0.005 (6)	0.003 (5)
C24	0.098 (9)	0.143 (11)	0.171 (12)	−0.018 (8)	−0.019 (8)	−0.016 (9)
C25	0.053 (4)	0.088 (5)	0.067 (5)	−0.008 (4)	0.009 (3)	−0.010 (4)
C26	0.058 (4)	0.084 (6)	0.062 (4)	0.000 (3)	0.005 (3)	−0.006 (3)
C27	0.074 (5)	0.116 (6)	0.060 (4)	−0.005 (4)	0.017 (4)	−0.001 (4)
C28	0.093 (5)	0.167 (8)	0.055 (4)	0.002 (11)	0.005 (3)	0.005 (10)
C29	0.066 (5)	0.084 (6)	0.090 (6)	0.019 (4)	−0.024 (4)	−0.002 (5)
C30	0.144 (9)	0.095 (7)	0.087 (7)	−0.009 (6)	0.025 (6)	0.001 (5)
C31	0.138 (11)	0.155 (12)	0.180 (13)	0.028 (9)	0.044 (9)	0.077 (10)
C32	0.205 (15)	0.130 (11)	0.206 (15)	0.041 (10)	0.076 (12)	0.083 (11)
C33	0.052 (4)	0.095 (6)	0.071 (5)	−0.010 (4)	−0.010 (4)	−0.013 (4)
C34	0.069 (5)	0.079 (5)	0.064 (4)	−0.002 (4)	0.008 (4)	−0.005 (4)
C35	0.086 (6)	0.151 (9)	0.073 (5)	0.011 (6)	−0.011 (5)	−0.043 (6)
C36	0.095 (8)	0.142 (10)	0.063 (6)	−0.008 (7)	0.007 (5)	−0.020 (6)
C37	0.080 (5)	0.059 (4)	0.068 (5)	−0.002 (4)	−0.003 (4)	0.009 (3)
C38	0.109 (7)	0.060 (5)	0.100 (7)	0.012 (5)	0.022 (6)	0.003 (5)
C39	0.089 (6)	0.076 (6)	0.107 (7)	0.017 (5)	−0.001 (5)	−0.004 (5)
C40	0.175 (12)	0.103 (8)	0.149 (11)	0.021 (8)	0.043 (10)	0.014 (8)
C41	0.077 (5)	0.090 (6)	0.051 (4)	0.006 (5)	0.007 (4)	0.007 (4)
C42	0.111 (7)	0.153 (9)	0.069 (5)	−0.058 (7)	−0.021 (5)	−0.003 (6)
C43	0.095 (7)	0.191 (12)	0.085 (7)	−0.007 (8)	−0.010 (6)	−0.015 (7)
C44	0.121 (10)	0.250 (17)	0.151 (12)	0.073 (11)	0.023 (8)	−0.027 (11)
C45	0.069 (4)	0.081 (5)	0.040 (3)	−0.014 (4)	0.003 (3)	0.008 (3)
C46	0.077 (5)	0.120 (7)	0.055 (4)	0.019 (5)	0.005 (4)	−0.004 (4)
C47	0.111 (7)	0.115 (7)	0.071 (5)	0.001 (6)	0.018 (5)	−0.021 (5)
C48	0.177 (12)	0.27 (2)	0.104 (8)	0.089 (13)	0.015 (8)	−0.011 (9)
C49	0.091 (5)	0.048 (4)	0.060 (4)	−0.011 (3)	0.018 (4)	0.013 (3)
C50	0.080 (5)	0.074 (5)	0.073 (5)	−0.018 (4)	0.014 (4)	−0.006 (4)
C51	0.136 (9)	0.205 (16)	0.098 (7)	−0.097 (11)	−0.006 (6)	0.036 (9)
C52	0.178 (13)	0.107 (13)	0.39 (2)	−0.054 (10)	0.145 (16)	−0.061 (14)

### Geometric parameters (Å, °)

**Table:**

Th—N1	2.480 (5)	C22—H22B	0.9700
Th—N2	2.465 (7)	C23—C24	1.487 (8)
Th—N3	2.488 (5)	C23—H23A	0.9700
Th—N4	2.490 (7)	C23—H23B	0.9700
Th—O1	2.618 (5)	C24—H24A	0.9600
Th—O2	2.563 (4)	C24—H24B	0.9600
Th—O4	2.557 (4)	C24—H24C	0.9600
Th—O5	2.570 (4)	C25—C26	1.521 (9)
Th—O7	2.615 (6)	C25—H25A	0.9700
Th—O8	2.524 (5)	C25—H25B	0.9700
S1—C1	1.596 (7)	C26—C27	1.554 (9)
S2—C2	1.613 (8)	C26—H26A	0.9700
S3—C3	1.626 (7)	C26—H26B	0.9700
S4—C4	1.599 (8)	C27—C28	1.513 (12)
O1—N5	1.264 (7)	C27—H27A	0.9700
O2—N5	1.288 (7)	C27—H27B	0.9700
O3—N5	1.232 (7)	C28—H28A	0.9600
O4—N6	1.274 (7)	C28—H28B	0.9600
O5—N6	1.263 (6)	C28—H28C	0.9600
O6—N6	1.202 (6)	C29—C30	1.508 (12)
O7—N7	1.258 (7)	C29—H29A	0.9700
O8—N7	1.263 (7)	C29—H29B	0.9700
O9—N7	1.243 (8)	C30—C31	1.557 (15)
N1—C1	1.167 (8)	C30—H30A	0.9700
N2—C2	1.172 (9)	C30—H30B	0.9700
N3—C3	1.163 (8)	C31—C32	1.487 (17)
N4—C4	1.156 (9)	C31—H31A	0.9700
N8—C13	1.495 (9)	C31—H31B	0.9700
N8—C5	1.516 (8)	C32—H32A	0.9600
N8—C17	1.517 (9)	C32—H32B	0.9600
N8—C9	1.522 (10)	C32—H32C	0.9600
N9—C29	1.504 (10)	C33—C34	1.521 (10)
N9—C25	1.521 (9)	C33—H33A	0.9700
N9—C21	1.531 (13)	C33—H33B	0.9700
N9—C33	1.532 (9)	C34—C35	1.496 (11)
N10—C37	1.506 (9)	C34—H34A	0.9700
N10—C41	1.514 (9)	C34—H34B	0.9700
N10—C45	1.514 (8)	C35—C36	1.491 (13)
N10—C49	1.521 (8)	C35—H35A	0.9700
C5—C6	1.528 (9)	C35—H35B	0.9700
C5—H5A	0.9700	C36—H36A	0.9600
C5—H5B	0.9700	C36—H36B	0.9600
C6—C7	1.505 (14)	C36—H36C	0.9600
C6—H6A	0.9700	C37—C38	1.502 (11)
C6—H6B	0.9700	C37—H37A	0.9700
C7—C8	1.545 (11)	C37—H37B	0.9700
C7—H7A	0.9700	C38—C39	1.526 (11)
C7—H7B	0.9700	C38—H38A	0.9700
C8—H8A	0.9600	C38—H38B	0.9700
C8—H8B	0.9600	C39—C40	1.517 (14)
C8—H8C	0.9600	C39—H39A	0.9700
C9—C10	1.507 (12)	C39—H39B	0.9700
C9—H9A	0.9700	C40—H40A	0.9600
C9—H9B	0.9700	C40—H40B	0.9600
C10—C11	1.507 (12)	C40—H40C	0.9600
C10—H10A	0.9700	C41—C42	1.513 (12)
C10—H10B	0.9700	C41—H41A	0.9700
C11—C12	1.518 (16)	C41—H41B	0.9700
C11—H11A	0.9700	C42—C43	1.500 (12)
C11—H11B	0.9700	C42—H42A	0.9700
C12—H12A	0.9600	C42—H42B	0.9700
C12—H12B	0.9600	C43—C44	1.465 (17)
C12—H12C	0.9600	C43—H43A	0.9700
C13—C14	1.516 (10)	C43—H43B	0.9700
C13—H13A	0.9700	C44—H44A	0.9600
C13—H13B	0.9700	C44—H44B	0.9600
C14—C15	1.531 (9)	C44—H44C	0.9600
C14—H14A	0.9700	C45—C46	1.500 (11)
C14—H14B	0.9700	C45—H45A	0.9700
C15—C16	1.546 (12)	C45—H45B	0.9700
C15—H15A	0.9700	C46—C47	1.515 (11)
C15—H15B	0.9700	C46—H46A	0.9700
C16—H16A	0.9600	C46—H46B	0.9700
C16—H16B	0.9600	C47—C48	1.485 (17)
C16—H16C	0.9600	C47—H47A	0.9700
C17—C18	1.517 (13)	C47—H47B	0.9700
C17—H17A	0.9700	C48—H48A	0.9600
C17—H17B	0.9700	C48—H48B	0.9600
C18—C19	1.517 (12)	C48—H48C	0.9600
C18—H18A	0.9700	C49—C50	1.530 (10)
C18—H18B	0.9700	C49—H49A	0.9700
C19—C20	1.51 (2)	C49—H49B	0.9700
C19—H19A	0.9700	C50—C51	1.513 (14)
C19—H19B	0.9700	C50—H50A	0.9700
C20—H20A	0.9600	C50—H50B	0.9700
C20—H20B	0.9600	C51—C52	1.496 (19)
C20—H20C	0.9600	C51—H51A	0.9700
C21—C22	1.525 (14)	C51—H51B	0.9700
C21—H21A	0.9700	C52—H52A	0.9600
C21—H21B	0.9700	C52—H52B	0.9600
C22—C23	1.520 (13)	C52—H52C	0.9600
C22—H22A	0.9700		
			
O1—Th—O2	49.51 (14)	C22—C21—H21A	108.7
O4—Th—O5	49.30 (13)	N9—C21—H21A	108.7
O7—Th—O8	49.44 (15)	C22—C21—H21B	108.7
N2—Th—N1	75.3 (2)	N9—C21—H21B	108.7
N2—Th—N3	74.4 (2)	H21A—C21—H21B	107.6
N1—Th—N3	143.89 (19)	C23—C22—C21	112.5 (8)
N2—Th—N4	142.8 (2)	C23—C22—H22A	109.1
N1—Th—N4	138.8 (2)	C21—C22—H22A	109.1
N3—Th—N4	76.5 (2)	C23—C22—H22B	109.1
N2—Th—O8	75.4 (2)	C21—C22—H22B	109.1
N1—Th—O8	70.55 (18)	H22A—C22—H22B	107.8
N3—Th—O8	83.21 (18)	C24—C23—C22	112.4 (9)
N4—Th—O8	123.3 (2)	C24—C23—H23A	109.1
N2—Th—O4	76.9 (3)	C22—C23—H23A	109.1
N1—Th—O4	119.51 (19)	C24—C23—H23B	109.1
N3—Th—O4	71.39 (16)	C22—C23—H23B	109.1
N4—Th—O4	72.3 (2)	H23A—C23—H23B	107.9
O8—Th—O4	146.4 (2)	C23—C24—H24A	109.5
N2—Th—O2	143.32 (19)	C23—C24—H24B	109.5
N1—Th—O2	72.00 (18)	H24A—C24—H24B	109.5
N3—Th—O2	127.36 (16)	C23—C24—H24C	109.5
N4—Th—O2	73.7 (2)	H24A—C24—H24C	109.5
O8—Th—O2	78.46 (15)	H24B—C24—H24C	109.5
O4—Th—O2	134.5 (2)	C26—C25—N9	114.7 (6)
N2—Th—O5	72.1 (2)	C26—C25—H25A	108.6
N1—Th—O5	71.26 (18)	N9—C25—H25A	108.6
N3—Th—O5	116.39 (16)	C26—C25—H25B	108.6
N4—Th—O5	101.48 (18)	N9—C25—H25B	108.6
O8—Th—O5	134.70 (15)	H25A—C25—H25B	107.6
O2—Th—O5	111.43 (14)	C25—C26—C27	108.8 (6)
N2—Th—O7	115.3 (2)	C25—C26—H26A	109.9
N1—Th—O7	108.01 (19)	C27—C26—H26A	109.9
N3—Th—O7	68.91 (18)	C25—C26—H26B	109.9
N4—Th—O7	73.88 (19)	C27—C26—H26B	109.9
O4—Th—O7	132.42 (16)	H26A—C26—H26B	108.3
O2—Th—O7	61.66 (16)	C28—C27—C26	111.0 (6)
O5—Th—O7	172.34 (16)	C28—C27—H27A	109.4
N2—Th—O1	131.50 (19)	C26—C27—H27A	109.4
N1—Th—O1	71.16 (18)	C28—C27—H27B	109.4
N3—Th—O1	144.92 (17)	C26—C27—H27B	109.4
N4—Th—O1	69.37 (18)	H27A—C27—H27B	108.0
O8—Th—O1	122.41 (16)	C27—C28—H28A	109.5
O4—Th—O1	90.27 (18)	C27—C28—H28B	109.5
O5—Th—O1	64.56 (15)	H28A—C28—H28B	109.5
O7—Th—O1	107.86 (17)	C27—C28—H28C	109.5
N5—O1—Th	96.0 (4)	H28A—C28—H28C	109.5
N5—O2—Th	98.0 (4)	H28B—C28—H28C	109.5
N6—O4—Th	97.9 (3)	N9—C29—C30	115.3 (8)
N6—O5—Th	97.6 (3)	N9—C29—H29A	108.5
N7—O7—Th	94.6 (4)	C30—C29—H29A	108.5
N7—O8—Th	98.8 (4)	N9—C29—H29B	108.5
C1—N1—Th	167.1 (6)	C30—C29—H29B	108.5
C2—N2—Th	172.2 (6)	H29A—C29—H29B	107.5
C3—N3—Th	170.5 (5)	C29—C30—C31	114.4 (9)
C4—N4—Th	159.1 (6)	C29—C30—H30A	108.7
O3—N5—O1	123.0 (6)	C31—C30—H30A	108.7
O3—N5—O2	120.5 (7)	C29—C30—H30B	108.7
O1—N5—O2	116.5 (6)	C31—C30—H30B	108.7
O3—N5—Th	177.2 (6)	H30A—C30—H30B	107.6
O1—N5—Th	59.4 (3)	C32—C31—C30	111.9 (13)
O2—N5—Th	57.1 (3)	C32—C31—H31A	109.2
O6—N6—O5	122.5 (6)	C30—C31—H31A	109.2
O6—N6—O4	122.6 (5)	C32—C31—H31B	109.2
O5—N6—O4	114.9 (5)	C30—C31—H31B	109.2
O6—N6—Th	176.1 (6)	H31A—C31—H31B	107.9
O5—N6—Th	57.8 (3)	C31—C32—H32A	109.5
O4—N6—Th	57.3 (3)	C31—C32—H32B	109.5
O9—N7—O7	123.5 (6)	H32A—C32—H32B	109.5
O9—N7—O8	119.3 (6)	C31—C32—H32C	109.5
O7—N7—O8	117.1 (6)	H32A—C32—H32C	109.5
O9—N7—Th	174.5 (5)	H32B—C32—H32C	109.5
O7—N7—Th	60.7 (4)	C34—C33—N9	115.8 (6)
O8—N7—Th	56.5 (3)	C34—C33—H33A	108.3
C13—N8—C5	105.9 (5)	N9—C33—H33A	108.3
C13—N8—C17	110.3 (6)	C34—C33—H33B	108.3
C5—N8—C17	110.5 (6)	N9—C33—H33B	108.3
C13—N8—C9	111.4 (6)	H33A—C33—H33B	107.4
C5—N8—C9	111.5 (6)	C35—C34—C33	112.0 (7)
C17—N8—C9	107.4 (6)	C35—C34—H34A	109.2
C29—N9—C25	112.6 (6)	C33—C34—H34A	109.2
C29—N9—C21	105.9 (6)	C35—C34—H34B	109.2
C25—N9—C21	110.8 (6)	C33—C34—H34B	109.2
C29—N9—C33	110.8 (6)	H34A—C34—H34B	107.9
C25—N9—C33	106.6 (5)	C36—C35—C34	116.5 (8)
C21—N9—C33	110.2 (6)	C36—C35—H35A	108.2
C37—N10—C41	109.9 (6)	C34—C35—H35A	108.2
C37—N10—C45	106.0 (5)	C36—C35—H35B	108.2
C41—N10—C45	110.4 (5)	C34—C35—H35B	108.2
C37—N10—C49	112.5 (5)	H35A—C35—H35B	107.3
C41—N10—C49	107.2 (5)	C35—C36—H36A	109.5
C45—N10—C49	110.9 (5)	C35—C36—H36B	109.5
N1—C1—S1	176.7 (7)	H36A—C36—H36B	109.5
N2—C2—S2	178.5 (7)	C35—C36—H36C	109.5
N3—C3—S3	179.7 (7)	H36A—C36—H36C	109.5
N4—C4—S4	177.1 (7)	H36B—C36—H36C	109.5
N8—C5—C6	115.4 (5)	C38—C37—N10	115.0 (6)
N8—C5—H5A	108.4	C38—C37—H37A	108.5
C6—C5—H5A	108.4	N10—C37—H37A	108.5
N8—C5—H5B	108.4	C38—C37—H37B	108.5
C6—C5—H5B	108.4	N10—C37—H37B	108.5
H5A—C5—H5B	107.5	H37A—C37—H37B	107.5
C7—C6—C5	110.6 (6)	C37—C38—C39	107.7 (8)
C7—C6—H6A	109.5	C37—C38—H38A	110.2
C5—C6—H6A	109.5	C39—C38—H38A	110.2
C7—C6—H6B	109.5	C37—C38—H38B	110.2
C5—C6—H6B	109.5	C39—C38—H38B	110.2
H6A—C6—H6B	108.1	H38A—C38—H38B	108.5
C6—C7—C8	110.8 (8)	C40—C39—C38	111.7 (8)
C6—C7—H7A	109.5	C40—C39—H39A	109.3
C8—C7—H7A	109.5	C38—C39—H39A	109.3
C6—C7—H7B	109.5	C40—C39—H39B	109.3
C8—C7—H7B	109.5	C38—C39—H39B	109.3
H7A—C7—H7B	108.1	H39A—C39—H39B	108.0
C7—C8—H8A	109.5	C39—C40—H40A	109.5
C7—C8—H8B	109.5	C39—C40—H40B	109.5
H8A—C8—H8B	109.5	H40A—C40—H40B	109.5
C7—C8—H8C	109.5	C39—C40—H40C	109.5
H8A—C8—H8C	109.5	H40A—C40—H40C	109.5
H8B—C8—H8C	109.5	H40B—C40—H40C	109.5
C10—C9—N8	114.8 (6)	C42—C41—N10	115.6 (7)
C10—C9—H9A	108.6	C42—C41—H41A	108.4
N8—C9—H9A	108.6	N10—C41—H41A	108.4
C10—C9—H9B	108.6	C42—C41—H41B	108.4
N8—C9—H9B	108.6	N10—C41—H41B	108.4
H9A—C9—H9B	107.6	H41A—C41—H41B	107.4
C9—C10—C11	111.0 (7)	C43—C42—C41	113.4 (9)
C9—C10—H10A	109.4	C43—C42—H42A	108.9
C11—C10—H10A	109.4	C41—C42—H42A	108.9
C9—C10—H10B	109.4	C43—C42—H42B	108.9
C11—C10—H10B	109.4	C41—C42—H42B	108.9
H10A—C10—H10B	108.0	H42A—C42—H42B	107.7
C10—C11—C12	113.1 (10)	C44—C43—C42	111.1 (10)
C10—C11—H11A	109.0	C44—C43—H43A	109.4
C12—C11—H11A	109.0	C42—C43—H43A	109.4
C10—C11—H11B	109.0	C44—C43—H43B	109.4
C12—C11—H11B	109.0	C42—C43—H43B	109.4
H11A—C11—H11B	107.8	H43A—C43—H43B	108.0
C11—C12—H12A	109.5	C43—C44—H44A	109.5
C11—C12—H12B	109.5	C43—C44—H44B	109.5
H12A—C12—H12B	109.5	H44A—C44—H44B	109.5
C11—C12—H12C	109.5	C43—C44—H44C	109.5
H12A—C12—H12C	109.5	H44A—C44—H44C	109.5
H12B—C12—H12C	109.5	H44B—C44—H44C	109.5
N8—C13—C14	116.7 (5)	C46—C45—N10	117.0 (6)
N8—C13—H13A	108.1	C46—C45—H45A	108.0
C14—C13—H13A	108.1	N10—C45—H45A	108.0
N8—C13—H13B	108.1	C46—C45—H45B	108.0
C14—C13—H13B	108.1	N10—C45—H45B	108.0
H13A—C13—H13B	107.3	H45A—C45—H45B	107.3
C13—C14—C15	110.5 (6)	C45—C46—C47	107.9 (7)
C13—C14—H14A	109.6	C45—C46—H46A	110.1
C15—C14—H14A	109.6	C47—C46—H46A	110.1
C13—C14—H14B	109.6	C45—C46—H46B	110.1
C15—C14—H14B	109.6	C47—C46—H46B	110.1
H14A—C14—H14B	108.1	H46A—C46—H46B	108.4
C14—C15—C16	114.0 (7)	C48—C47—C46	115.9 (9)
C14—C15—H15A	108.8	C48—C47—H47A	108.3
C16—C15—H15A	108.8	C46—C47—H47A	108.3
C14—C15—H15B	108.8	C48—C47—H47B	108.3
C16—C15—H15B	108.8	C46—C47—H47B	108.3
H15A—C15—H15B	107.7	H47A—C47—H47B	107.4
C15—C16—H16A	109.5	C47—C48—H48A	109.5
C15—C16—H16B	109.5	C47—C48—H48B	109.5
H16A—C16—H16B	109.5	H48A—C48—H48B	109.5
C15—C16—H16C	109.5	C47—C48—H48C	109.5
H16A—C16—H16C	109.5	H48A—C48—H48C	109.5
H16B—C16—H16C	109.5	H48B—C48—H48C	109.5
N8—C17—C18	115.7 (7)	N10—C49—C50	114.2 (5)
N8—C17—H17A	108.3	N10—C49—H49A	108.7
C18—C17—H17A	108.3	C50—C49—H49A	108.7
N8—C17—H17B	108.3	N10—C49—H49B	108.7
C18—C17—H17B	108.3	C50—C49—H49B	108.7
H17A—C17—H17B	107.4	H49A—C49—H49B	107.6
C19—C18—C17	112.7 (9)	C51—C50—C49	105.5 (6)
C19—C18—H18A	109.1	C51—C50—H50A	110.6
C17—C18—H18A	109.1	C49—C50—H50A	110.6
C19—C18—H18B	109.1	C51—C50—H50B	110.6
C17—C18—H18B	109.1	C49—C50—H50B	110.6
H18A—C18—H18B	107.8	H50A—C50—H50B	108.8
C20—C19—C18	112.0 (10)	C52—C51—C50	111.4 (9)
C20—C19—H19A	109.2	C52—C51—H51A	109.4
C18—C19—H19A	109.2	C50—C51—H51A	109.4
C20—C19—H19B	109.2	C52—C51—H51B	109.4
C18—C19—H19B	109.2	C50—C51—H51B	109.4
H19A—C19—H19B	107.9	H51A—C51—H51B	108.0
C19—C20—H20A	109.5	C51—C52—H52A	109.5
C19—C20—H20B	109.5	C51—C52—H52B	109.5
H20A—C20—H20B	109.5	H52A—C52—H52B	109.5
C19—C20—H20C	109.5	C51—C52—H52C	109.5
H20A—C20—H20C	109.5	H52A—C52—H52C	109.5
H20B—C20—H20C	109.5	H52B—C52—H52C	109.5
C22—C21—N9	114.4 (5)		
